# To have your citizen science cake and eat it? Delivering research and outreach through Open Air Laboratories (OPAL)

**DOI:** 10.1186/s12898-016-0065-0

**Published:** 2016-07-22

**Authors:** Poppy Lakeman-Fraser, Laura Gosling, Andy J. Moffat, Sarah E. West, Roger Fradera, Linda Davies, Maxwell A. Ayamba, René van der Wal

**Affiliations:** 1 Centre for Environmental Policy, Imperial College London, South Kensington, London, SW7 1NA UK; 2 Forest Research, Alice Holt Lodge, Farnham, Surrey GU10 4LH UK; 3 Stockholm Environment Institute, University of York, Heslington, York, YO10 5DD UK; 4 Department for the Natural and Built Environment, Faculty of Development and Society, Sheffield Hallam University, Sheffield, S1 1WB UK; 5 Aberdeen Centre for Environmental Sustainability, School of Biological Sciences, University of Aberdeen, Aberdeen, AB24 3UU UK

**Keywords:** Citizen science, Evaluation framework, Lessons learned, OPAL, Outputs, Outreach, Public participation in scientific research, Research, Trade-off, Volunteers

## Abstract

**Background:**

The vast array of citizen science projects which have blossomed over the last decade span a spectrum of objectives from research to outreach. While some focus primarily on the collection of rigorous scientific data and others are positioned towards the public engagement end of the gradient, the majority of initiatives attempt to balance the two. Although meeting multiple aims can be seen as a ‘win–win’ situation, it can also yield significant challenges as allocating resources to one element means that they may be diverted away from the other. Here we analyse one such programme which set out to find an effective equilibrium between these arguably polarised goals. Through the lens of the Open Air Laboratories (OPAL) programme we explore the inherent trade-offs encountered under four indicators derived from an independent citizen science evaluation framework. Assimilating experience from the OPAL network we investigate practical approaches taken to tackle arising tensions.

**Results:**

Working backwards from project delivery to design, we found the following elements to be important: ensuring outputs are fit for purpose, developing strong internal and external collaborations, building a sufficiently diverse partnership and considering target audiences. We combine these ‘operational indicators’ with four pre-existing ‘outcome indicators’ to create a model which can be used to shape the planning and delivery of a citizen science project.

**Conclusions:**

Our findings suggest that whether the proverb in the title rings true will largely depend on the identification of challenges along the way and the ability to address these conflicts throughout the citizen science project.

## Background

Citizen science, in all its diverse manifestations, is a burgeoning field of scientific endeavour. Considered by some to be part of ‘public participation in scientific research’ (PPSR) [1], it is a branch of contemporary science which is used to describe a vast array of activities. It spans subjects from identifying simple morphological classifications of galaxy shapes [2]; to competing in a multiplayer online game to discover protein structure models [3]; to field-based monitoring of commercial poachers in the Congo basin rainforest by Mbendjele hunter-gather communities [4]. As such, the umbrella term has come to mean different things to different people, but is now defined in the Oxford English Dictionary as “scientific work undertaken by members of the general public, often in collaboration with or under the direction of professional scientists and scientific institutions” [5].

Data collection by amateurs has, in many cases, pre-dated paid scientific professions [6, 7]; however, the modern movement of citizen science is still in its infancy. It is a term used to describe a new approach to scientific investigation that, riding on the wave of technology, is open to a broad audience, rather than a wealthy few ‘gentleman scientists’ [8]. The term ‘citizen science’ was coined independently in the mid-1990s by Rick Bonney in the US [9] and Alan Irwin in the UK [10]. Citizen science to Bonney was concerned with science communication and public participation in science; whereas for Irwin, the focus was to enhance the accessibility of science policy processes to the public [11]. These descriptions broadly align with two academic movements that have influenced and sculpted the discipline of citizen science: ‘public understanding of science and technology’ (PUST) which enhances public knowledge and acceptance of science; and ‘public engagement in science’ (PES) which draws on participatory democratic ideals in scientific research, practice and policy [12]. Paralleling drivers of PUST and PES, investigations into citizen science project goals [13], reasons for participants to become involved [14] and benefits that projects yield [15] reveal two broad themes—outreach and research. ‘Outreach’ (i.e. an effort to bring services or information to people [16]) includes potential benefits to individuals [through providing learning and training opportunities (e.g. about the natural world)]; benefits to the scientific community [such as promoting science as a worthy cause or expanding awareness of new application areas (e.g. astronomy)]; or benefits to society [such as changing public behaviour (e.g. to prevent spread of invasive species)]. ‘Research’ (i.e. detailed study of a subject, especially in order to discover (new) information or reach (new) understanding [17]) not only includes potential benefits for scientists (in gathering, analysing and interpreting large data sets); but also benefits for policy makers or for society as a whole (via collectively gathering evidence and acquiring knowledge from non-traditional sources).

Some suggest [14] that citizen science “must place equal emphasis on scientific outcomes and learning outcomes” (p. 313) and many see striving to obtain both goals as a ‘win–win’ situation [18, 19], where increased participation in science yields enhanced learning opportunities and advanced research outcomes [14]. Trade-offs can however be experienced. For example, creating a project which yields rigorous data sets through complex protocols can be a barrier, potentially limiting the number and retention of participants [20, 21]; or alternatively striving for strong outreach benefits whilst paying little attention to accuracy can potentially lead to datasets of unknown quality and limit their value [22, 23]. Dickinson and Bonney [13] studied 80 projects and asked developers to assign a weight to the goals of the project. They found a significant negative relationship between the goals of education and scientific research, suggesting that investment in one compromised investment in the other. In a similar vein, Zoellick et al. [14] found that the more the students in a classroom benefited from the citizen science experience the less the scientist benefited and vice versa. Given this recognised trade-off, is it possible for citizen science projects to successfully achieve both aims, and if so how? Heeding the advice of one citizen science practitioner [11]—“next to all the enthusiastic endorsements of the many undoubted positive aspects of CS [citizen science] we also keep in mind the limits of what CS can realistically achieve, and keep up a conversation about how to address the limitations of CS” (p.118)—we investigate a programme that aims to balance these goals at a broad scale, Open Air Laboratories (OPAL).

## Methods

### Case study

**Fig. 1:**
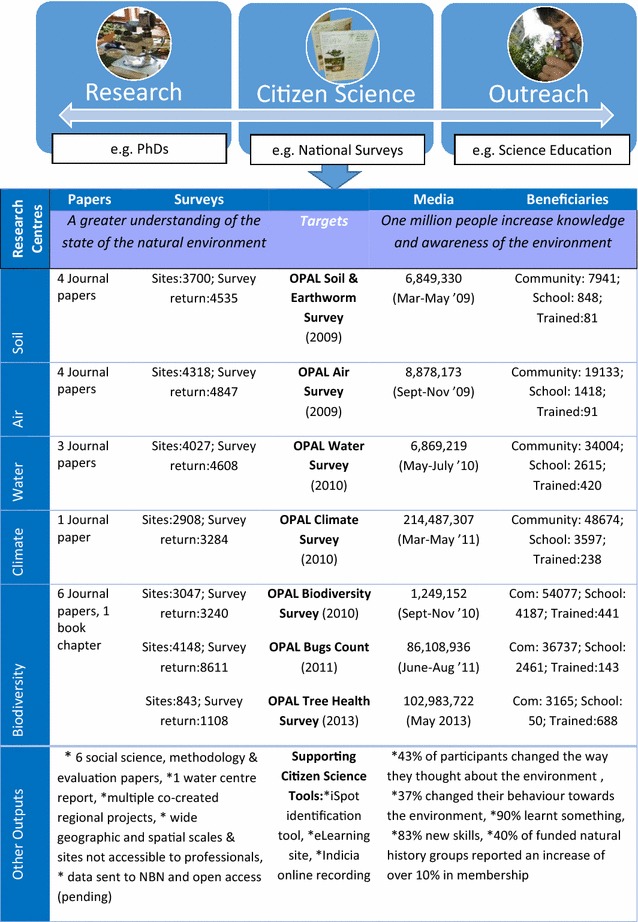
OPAL operations span outreach to research goals and utilise OPAL national surveys to deliver citizen science elements of the public engagement in science programme. One survey was produced per research centre, except Biodiversity where three were produced. The number of papers (i.e. where OPAL is mentioned in acknowledgements) and number of sites monitored (i.e. unique latitude and longitude) are displayed for each science theme. Beneficiary figures were obtained from OPAL monthly evaluation forms between April 2010–December 2013 and media figures were based on circulation and web hits to online articles over the period which the survey was at its most popular. ‘Other outputs’ record citizen science tools and output beyond OPAL surveys [24–26]

OPAL is a UK-based public engagement in science programme which utilises citizen science to deliver both outreach and research. It does so from local to national scales, aiming to create ‘citizen science for everyone’ regardless of age, background or ability. Initiated in 2007 by Imperial College London, the programme was funded with £11.7 million (with three later awards in 2010, 2011 and 2013 increasing this total to £17.4 million) by the Big Lottery Fund (BLF). The original phase of OPAL represented a network of 15 organisations including: ten universities, one natural history museum, one educational organisation, one biological recording organisation, a parks consortium and an environmental government department. Initially operating across England (the period on which this review focusses) and in 2014 expanding to Scotland, Wales and Northern Ireland, the consortium aimed to place scientists into communities to share knowledge and engage individuals in field-based research [7]. It did this through a network of Community Scientists (science engagement staff), project leaders (academics based in each of the institutions), PhD students (based in nine geographically designated regions across the country) and external organisations (who provided an advisory role for specific activities). Five ‘research centres’ (academic research consortia) were assimilated from individuals from partner organisations on the topics of Air, Water, Climate, Soil and Biodiversity. OPAL’s operations traversed the gradient from research approaches to outreach approaches involving professional researchers and citizen scientists to varied extents (Fig. 1). The network took a number of approaches, from delivering citizen science through online tools [24–26] to local co-created citizen science projects. However, the primary mechanism used was the series of seven environmental national surveys led by each of the aforementioned research centres and shaped by other relevant external organisations. These were the: OPAL Soil and Earthworm Survey, OPAL Air Survey, OPAL Water Survey, OPAL Climate Survey, OPAL Biodiversity Survey, OPAL Bugs Count and OPAL Tree Health Survey. Each survey was made freely available to participants (either in hard copy format or through digital downloads) to ensure inclusivity and consisted of a pack which contained everything required to conduct the survey. For example, the OPAL Soil and Earthworm Survey pack contained a field notebook (rationale for conducting the research and recording sheets to collect data on site characteristics, soil properties and earthworms), a field guide (earthworm identification guide and survey steps) and equipment (pH strips, magnifier, vinegar and mustard). All surveys aimed to raise awareness about key environmental issues and scientific methodologies; and generate data on selected scientific questions, e.g. species distributions, changing environmental conditions or the impact of urbanisation on biodiversity.

The programme was designed with the dual purpose of “bringing scientists and communities together to deliver a research programme focused on three environmental themes: loss of biodiversity, environmental degradation and climate change” (research) and “motivating outdoor exploration and providing participants with the knowledge, skills and confidence needed to study nature” (outreach) [27]. The research aim was driven by the Conventions on Climate Change and Biodiversity [28] and the crisis in taxonomy [29]. OPAL’s outreach objective was driven by a decline in outdoor learning in the UK [30] and a call for programmes addressing education and engagement of local communities by the BLF [31] who award money to projects that improve health, education and the environment [7]. Targets were set for both research and outreach, the former being driven by the broad aim to ‘achieve a greater understanding of the state of the natural environment’ and the latter having the specific aim for ‘one million people to increase knowledge and awareness of the environment’. These targets were monitored throughout the programme (Fig. 1). For research outputs, over 230,000 packs were distributed to the public and surveys were submitted which translated to 25,000 field sites being sampled [27]. The aim was to send all appropriate species level data to the National Biodiversity Network with the eventual target to make all data open access. In addition, academic journal papers were produced reporting on ecological results from the national surveys [19, 32–35], specific regional research [36–39], methodological research [19, 32, 34, 40–42], effective working practices [43, 44], and perceptions of citizen science amongst scientists [11]. All outreach targets were reached and in many cases exceeded with a total of over 850,000 direct beneficiaries (i.e. distinct learning experiences through events, lessons and community presentations) and almost 1.7 million website hits reached between February 2008 and November 2013 (Fig. 1). Online questionnaires filled out by a sample of participants following the data entry of national surveys revealed outcomes such as awareness raising, behaviour change, learning outcomes and impact on learning pathways (Fig. 1). Because of this broad approach and range of outputs, OPAL provides a useful case study through which to explore the trade-offs inherent in dual-aim programmes and investigate the factors which helped deliver the citizen science elements of the programme.

### Data

Monitoring and evaluation has been conducted since OPAL was initiated (2008–2013 reported here) in order gather evidence of the number of participants in OPAL activities (referred to as ‘beneficiaries’) for the funder (BLF) and to capture experiences of those involved in OPAL because of its large and distinct modus operandi so that lessons could be learned and communicated to benefit future citizen science endeavours. One study [15] suggested the importance of evaluating a citizen science programme not only in terms of its scientific outputs (which tend to be confined to hypothesis-led research and mainly positive data outcomes) but also from experiences of programme staff (who were well placed to comment upon the developmental process of the project and report on problems encountered). This investigation therefore not only employs quantitative information (i.e. beneficiary numbers) but also uses qualitative material on lessons learned from staff and participants.

**Fig. 2:**
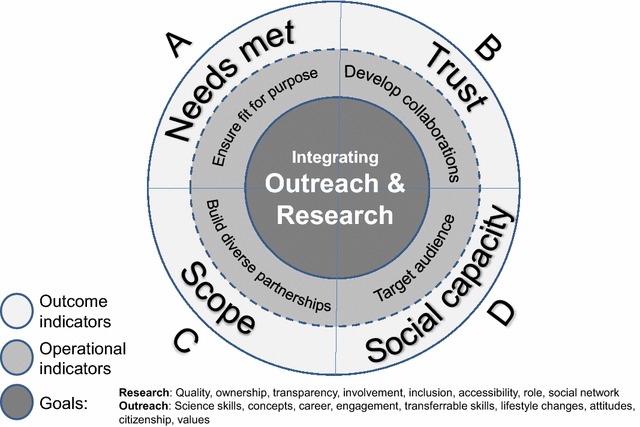
A model demonstrating the relationship between project goals, operations and outcomes, the indicators of which contribute towards a successful citizen science project. Goal and outcome indicators are derived by research conducted by Haywood and Besley [12, 45] and operational indicators are derived from lessons learned throughout the OPAL programme

Quantification of the extent to which people engaged with the OPAL programme was obtained from monitoring forms which the original 15 OPAL partners returned on a monthly basis to the programme management team. They primarily reported on the number of members of the public who participated in OPAL activities and also the schools and community, voluntary and statutory organisations they had worked with. A total of 1107 monitoring forms were collected from OPAL partners between 2008 and 2013, and these formed the basis of the quantitative data reported. At the end of every project year, partners were also required to report on their activities over the past 12 months. As part of these, partners were asked to answer the following questions: ‘What are the five most important lessons learned since your project began?’ (Year 4) and ‘What are the five most important lessons learned from delivering your project?’ (Year 5). A total of 60 annual reports formed the basis of our qualitative data. A log of ‘lessons learned’ was also maintained by OPAL management. This formed additional data that were qualitatively appraised. Qualitative data was used to deepen understanding gained from formal quantitative reporting to explore trade-offs and potential solutions for balanced operations. Key themes were drawn out of the evidence, and text fragments identified as belonging to each category were brought together and assimilated into the four themes presented in Table 1 and Fig. 2. In the presentation of our findings we use quotes to illustrate both widely shared and minority views, with explicit indication of the latter.

**Table 1:** Textual data obtained through monthly monitoring by OPAL staff which inform operational indicators

	Outcome indicator & trade-off	Operational indicator	Key questions	Example quotations providing evidence for operational indicators
A	*Needs met* Trade-off: *Outreach gets in the way of research and research gets in the way of outreach?*	Ensure outputs are *fit for purpose*	Is the project *designed* and *monitored* appropriately to ensure outreach and research of an appropriate quality?	*“Activities are best split into bite-sized chunks that can be done singly with less interested individuals and in multiples with the more interested.” *“resources …have to be concise, visually interesting, and different.” *“important not to ask the public to run before they can walk” *“focus on single or small set of bio-indicator species addressed the challenge of identification expertise, whilst proving less daunting and more empowering for the volunteer” *“citizen science projects must (1) provide sufficient training to ensure data are collected accurately, and (2) regularly monitor and screen incoming data to ensure continued accuracy.” *“Planning a programme of evaluation from the outset is very useful and ensures that it is ingrained in everybody’s thinking form the outset.” *“Qualitative and quantitative evaluation are both valuable”
B	*Trust * Trade-off: *Build a reputation with partners or participants?*	Develop *strong collaborations*	Is there adequate *buy*-*in* from partners and is *feedback* maintained?	*“Important to get appropriate buy-in from scientists who should/will be involved… especially in scientific disciplines where citizen science is new, novel or perceived as threatening.” *“the key to making links with existing community groups is finding and highlighting ways in which it is possible to work together”.*“The willingness of people to initially engage with the OPAL project appears to be enhanced when they are introduced to the project through face-to-face contact. Once initial engagement is made, many continue to request survey packs and information about events etc. in a more remote manner (telephone, email).” *“The surveys need to give instant results that people can relate to the quality of their local environment. The water survey was particularly good for this as the Pond Health score gave people a measure of their pond’s water quality”
C	*Scope* Trade-off: *Jack of all trades, master of none?*	Build a sufficiently *diverse partnership*	Is there appropriate *expertise* within the programme?	* “My main take away lessons from my time as a Community Scientist are that you need to share your passion for the natural world.” *“Community Scientists were involved with the development of all 7 national surveys, e.g. from testing the survey with local communities to providing feedback on the final survey materials…This resulted in the development of surveys which the general public could participate in/contribute successfully to, as well as generating meaningful scientific data for OPAL.” *“it is vital to obtain formal agreement from senior managers of partner organisations to provide the resources (especially time) to fulfil their obligations.” *“Initially it was difficult to build up relationships with schools or to have anything other than one-off interactions.” *“Academics are not usually involved in this scale of public outreach. It proved to be very rewarding on many fronts”
D	*Social capacity* Trade-off: *Who is contributing and to what extent?*	*Target audience*	Which *sectors of society* are considered and is *technology* integrated appropriately?	* “Genuinely hard to reach community groups require large commitments of time and energy to build up relationships to the level where outreach can be delivered successfully.” *“because once relocated to England, and especially for second generation and the younger generation, human activities are no longer seen as part of any ecosystem function.” *“Issues of inclusivity have to be faced professionally” *“Technical developments intended to be a major part of a public engagement project need to be carefully planned for, well in advance, to ensure that they can be taken up effectively by participants.” *“People have also increasingly moved towards using mobile and tablet devices since OPAL first started and as this tech is now part of their everyday lives, we need to respond to this demand.” *“Use of digital technology (e.g. social media) offers us a way to reach out to this audience in the spaces that they already frequent, at very little expense to us”

These indicators inform the practical considerations when addressing trade-offs

## Investigation and management of trade-offs across the outreach-research spectrum

Haywood and Besley [12, 45] developed a set of standards for PPSR projects (of which citizen science is a part), deeming four key dimensions central to the integration of ‘education outreach’ (broadly aligned with our definition of outreach) and ‘participatory engagement’ (broadly aligned with our definition of research) traditions. The findings suggested that a successful project should: *A. meet the needs* of all stakeholders*, B. foster trust and confidence* among stakeholders and in science*, C. broaden scope and influence*, and *D.* build *social capacity* to respond to ecological challenges. We use their conceptualisation as a theoretical model to: (1) reveal the key trade-offs between delivering research and outreach within the large-scale OPAL programme; and (2) discuss operational considerations to manage the identified trade-offs. Based on this exploration, we propose an evaluation framework (Fig. 2), which builds on Haywood and Besley’s [45] standards (‘outcome indicators’) and pairs these with effective working practices (‘operational indicators’) that could address trade-off challenges.

### Revealing key trade-offs within OPAL

#### A. Needs met

The first (outcome) indicator which Haywood and Besley [45] identify in their assessment framework is the “degree to which the products generated (intellectual or material) meet the legitimate needs and expectations of participants [all stakeholders involved]” (p. 5). A key trade-off emerged from OPAL across the research outreach spectrum in this context of ‘needs met’: *outreach gets in the way of science* (i.e. science needs being compromised) *and science gets in the way of outreach* (i.e. outreach needs being compromised) (Table 1). Firstly, regarding outreach getting in the way of science, one scientist collaborator commented that “experience from OPAL [Tree Health Survey] and Sylva Tree Watch surveys suggest that lay involvement in tree health surveillance is at best only partially successful from a scientific perspective.” While every effort was made to ensure scientific thoroughness, data acquisition could be compromised because “groups adopted a pick-and-mix approach to the different tasks and activities in the survey. This is driven by the fact that every group has their own range of ages and abilities, levels of scientific knowledge, plus unique time and logistical constraints.” Next, the return rates for the number of OPAL surveys distributed compared to completed surveys submitted was around 10 %, partially because “after a field session people did not always want to sit down and upload all the data at a computer”. While anecdotally this may be a good level compared to industry standards, such return rates may not be acceptable in terms of the efficient utilisation of scientific resources (depending upon whether hard copies are used or packs downloaded). Perhaps one of the most fundamental debates in citizen science is the preconception that it is either possible to collect large quantities of data which is of questionable accuracy or small quantities of highly accurate data. The use of photographs and apps for mass collection of verifiable data (for example the UK Ladybird Survey and OPAL Species Quest [46, 47]) can help here, but where this (or another quality monitoring technique) was not effectively utilised within OPAL, scientists found that “data are patchy and of poor quality”. The scientists were not the only ones who expressed concern about generating usable data: “it would appear that most participants will try to undertake the survey to the best of their ability […and] generally appear to be concerned about data quality and some even decline to submit their data as a result.” Rigorous, complete data sets are required for science so are outputs being compromised with the citizen science approach?

Secondly, OPAL staff regularly commented that “science (activity) may interfere or get in the way of actual involvement [i.e. learning by doing]”. OPAL’s focus on fixed packs, developed to simultaneously generate outreach and data to address specific questions, meant that outreach opportunities were sometimes missed because the focal landscape components of some packs (e.g. ponds, hedges) could be in short supply: “while the survey was written for anyone to be able to participate, one of the biggest hurdles has been for people to find a publically accessible pond.” The one-size-fits-all approach for generating useful data created further challenges: “OPAL designed each survey to appeal to an age range of 13–14 years. We felt this would also provide a valuable experience for newcomers and those without previous knowledge of the topics. Many people used the surveys with young children and they were not suitable, particularly without high levels of adult supervision”. While it may seem that research may initially reduce the impact of outreach, “there is a need to be realistic in expectations of immediate payback - perhaps the greater return (in terms of developing scientific interest) will come much later if scientific interest is sustained in those who take part”. Is it therefore possible to maintain high quality outreach outputs, and if so how?

#### B. Trust and confidence

A second outcome indicator on which citizen science programmes should be evaluated according to Haywood and Besley [45] is the “degree to which the project fosters general trust, confidence, and respect among project participants and in science” (p.5). A key trade-off emerged from OPAL across the research outreach spectrum in the context of ‘trust and confidence’: *Do you put resources into building a reputation with partners (research) or participants (outreach)?* (Table 1). As one partner commented, “developing collaboration requires more time and support than originally envisaged and has been a major challenge.” Firstly, building trust and respect between organisations is draining on resources: “many such relationships, particularly between academia and local and national government and other organizations have developed but required considerable effort.” It can be difficult to plan for when building relationships as “it was challenging to define an enabling project on the basis of the expected needs of a new and unknown partnership.” Working closely with other organisations can also create challenges: “Elements of our project have been reliant upon OPAL partner organisations for their successful delivery, and in some cases unforeseen logistical problems have prevented them taking place or greatly increased the workload involved.” A ‘one-size-fits-all’ approach may not work when building relationships with groups because organisations vary in the objectives they have to meet and their expectations from the working relationship. For example, “some who lent expertise to OPAL felt that their society should be paid at a consultancy rate for it. At the other end of the spectrum, others felt that claiming travel and subsistence funds when they attended events was wrong because they were doing it for the love of it and didn’t feel comfortable reclaiming the money.” Secondly, building the confidence of participants is integral to yielding social benefits as well as scientific outputs: “the real satisfaction of the [Community Scientist] role is watching others’ skills and confidence grow” and “we have observed people gain the confidence to not only lead surveys in their own communities but to realise that resources such as iSpot [a species identification website] are accessible and engaging.” On top of this, building trust and confidence between practitioners and participants is fundamentally important: “if survey coordinators do not find it possible to trust potential participants to undertake the surveys in the spirit they are intended then the voluntary participant approach is probably not the right approach for the task.” This raises an important point, namely that citizen science is not always appropriate for all types of research questions (see [41] for a tool to help practitioners decide whether citizen science is appropriate). Is it an ‘either/or’ situation, or can resources, trust and confidence be built with both partners and participants, and if so, how?

#### C. Scope

According to Haywood and Besley [45] projects should also be evaluated on the “degree to which products generated (intellectual or material) impact broader social, economic, or environmental systems and relevant policy (e.g. local laws and procedures, national standards, corporate practices)” (p. 5). A key trade-off emerged from OPAL across the research outreach spectrum in the context of ‘*scope’: jack of all trades and master of none* (i.e. are both outreach and research needs being compromised)*?* (Table 1). OPAL aimed to create “science-society-policy interactions which would lead to a more democratic research, based on evidence-informed decision making”, but in doing so does the programme and its staff become generally effective across multiple sectors but not outstanding in any of them? For example in policy, OPAL Tree Health Survey is referenced in a parliamentary ‘POSTNOTE’ paper as an example of a project which contributes towards Defra’s Tree Health and Biosecurity Action Plan [48]; in education, OPAL activities are thought to have had an influence on improving science GCSE grades [27]; and in research, the OPAL Air Survey found that citizens access new areas, previously underexplored by professional scientists [32]. The trade-off lies potentially in the progress in each of these areas. Have efforts into realising impact in society meant that not as much was being achieved in research? To consider this, Fig. 1 demonstrates the relative balance between the impact in each sector. One scientist collaborator who was sceptical about the usefulness of the scientific output generated commented that it is “important for national biosecurity that a formal assessment of citizen science impact is made sooner rather than later or not at all so that alternative strategies be rolled out—the subject is too important for prevarication, political correctness or fudging.” Indeed, the influence of citizen science on academia for example can look minimal when assessed using recognised institutional indices (i.e. publication output) [43]. While staff may have been making inroads in enriching learning experience of participants and enhancing awareness, all that is recorded is the ‘pounds per paper’ so that a large investment of money looks squandered against academic output. This can put a strain on the staff delivering these projects “it’s several roles rolled into one… it’s essentially…two part time jobs…, they’re not, they’re two full time jobs to do them properly” [49]. This was also reflected in the feedback that was obtained suggesting that there was “an understandable reticence for some sectors of the scientific profession to engage in CS—‘what will this add to my project,—or my career?’” Therefore when measured against traditional systems, whether that be outreach or research—the amount of impact may not be as large as concentrating on one discipline. This, of course, is not accounting for cumulative impacts of the two, measurement scales for which may not fully exist yet. Paradigms are however shifting as the Research Excellence Framework (the system used to assess the quality of research in UK higher education institutions [50]) increasingly recognises *impact* (“‘reach and significance’ of impacts on the economy, society and/or culture that were underpinned by excellent research” [51]). While the majority is focussed on outputs (e.g. academic papers), impact now carries a weighting of 20 % and citizen science projects are now being recognised as ideal case studies and excellent ways to generate publicity for institutions. Is it therefore possible to have a recognised impact across a range of sectors (academia, education and policy) rather than just one, and if so, how?

#### D. Social capacity

A final dimension Haywood and Besley [45] propose citizen science projects to be evaluated on is the “degree to which the project influences the capacity of communities/social groups to respond to social or ecological challenges, negotiate conflicts, and develop solutions” (p.5). A key trade-off emerged from OPAL across the research-outreach spectrum in the context of ‘social capacity’: *who is contributing* (well informed public-supporting research needs, or all sectors of society-supporting outreach) *and to what extent* (high level of involvement of scientists-potentially benefiting research, or high levels of involvement of the public-potentially benefiting outreach)? (Table 1). OPAL’s remit was to work with hard to reach communities and staff commented: “the deepest interactions are undoubtedly the most rewarding, but they are also the most resource intensive”. It was therefore a conundrum to many Community Scientists between reaching as many beneficiaries to support them in producing high quality science and reaching sectors of society that may not have the opportunity to access these opportunities. One of OPAL’s collaborators suggested, perhaps controversially, that given the “data are not from a random sample anyway, does it matter scientifically if some sections of society are excluded?”

Within citizen science there is a spectrum of engagement from contributory (designed by scientists, public contribute data) to collaborative (designed by scientist, public contribute to design, data and analysis) to co-created (scientists and public work together on all aspects of process) projects [1], and typically a particular project will focus on one approach. For OPAL, the primary focus has been on allocating resources to the ‘contributory’ style national survey with far less emphasis being placed on the citizen- driven ‘co-created’ studies. Although at a regional level Community Scientists worked with local groups to develop research important to these groups (on topics ranging from invasive crayfish monitoring to hedgehog tracking), this was a small element of the programme. There was a call to move towards this end of the spectrum with one Community Scientist suggesting “to ensure successful delivery of the programme the community has to help shape and plan the project aims and objectives (i.e. bottom-up)”. Those OPAL staff who operated that way suggested that through involving participants from the beginning on issues that are important to them, projects were more likely to recruit participants and collect data that would be used, if only locally. Others within the network held alternative opinions: “I have mixed views on co-creation. It seems to be the scientists who are saying that this is what people want. This may be true sometimes and in some situations but not always. Sometimes citizens are content to let the experts develop the project and to assist by data gathering. I have even heard negative attitudes to greater involvement: we do not have the skills/that is what experts are for/we are too busy with our own jobs etc.” Is it therefore too challenging to take multiple approaches when planning the level at which participants get involved and if so what is the important factor to consider to support social capacity?

### Managing trade-offs in OPAL

Is it possible to address these trade-offs and deliver outputs useful for both outreach and research? Qualitative data generated by OPAL staff and participants (Table 1) contribute to an understanding of how to practically confront each of the four challenges under the outcome indicators set out by Haywood and Besley [45]. These effective working practices were used to develop four operational indicators in order to provide a potential solution to a number of the trade-offs raised: ensuring outputs are fit for purpose, developing strong collaborations, building a sufficiently diverse partnership and targeting specific audiences.

#### A. Fit for purpose (to ensure needs are met)

Given that the first of the trade-offs was: *outreach gets in the way of science and science gets in the way of outreach*, there is a clear requirement for the products generated (resources, events, data etc.) to be *fit for purpose* for both research and outreach. Two essential points feed into this: the quality of the products need to be *designed* appropriately for their intended use and *monitoring* needs to be built in to track quality of the data and outreach (Table 1).

From the perspective of outreach, outputs that are fit for purpose attract participants, maintain their enthusiasm and ensure the science content is understandable. Resources (e.g. the OPAL packs) should clearly communicate an aim, be visually interesting, understandable and sufficiently flexible in their use (Table 1). To explore this theme in more detail, we focus on the creation of scientific outputs that are fit for purpose. To ensure that the data collected are of a quality which is usable for the intended purpose, methodological design is key, training should be considered, technology used appropriately, monitoring of accuracy implemented and analysis techniques tailored to the data utilised (Table 1). One contributor noted that “it was in fact these quite substantial worries about data quality that drove them [practitioners] to be methodologically innovative in their approach to interpreting, validating and manipulating their data and making sure that the science being produced was indeed new, important and worth everyone’s time.” In many cases, survey leaders thought carefully about balancing the needs of participants and data users. For example in the Bugs Count, the first activity asked the public to classify invertebrates into broad taxonomic groups (which were easier to identify than species) and the second activity asked participants to photograph just six easy-to-identify species. Participants therefore learned about what features differentiate different invertebrate groups whilst collecting valuable verifiable information on species distribution (e.g. resulting OPAL tree bumblebee data were used in a study comparing skilled naturalist and lay citizen science recording [52]). Data quality monitoring was conducted to varying degrees between surveys. The Water Survey [34] for example, integrated training by Community Scientists, identification quizzes, photographic verification, comparison to professional data and data cleaning techniques. Survey leads on the Air Survey [32] compared the identification accuracy of novice participants and expert lichenologists and found that for certain species of lichen, average accuracy of identification across novices was 90 % or more, however for others accuracy was as low as 26 %. Data with a high level of inaccuracy were excluded from analysis and “this, together with the high level of participation makes it likely that results are a good reflection of spatial patterns [of pollution] and abundances [of lichens] at a national [England-wide] scale” [32]. For the Bugs Count Survey, information on the accuracy of different groups of participants was built into the analysis as a weight, so that data from groups (age and experience) that were on average more accurate, contributed more towards the statistical model [19]. This exemplifies that if data quality is being tracked, and sampling is well understood, then a decision can be made by the end user about which datasets are suitable for which purpose.

#### B. Develop strong collaborations (to build trust and confidence)

To tackle the second key trade-off—*building a reputation with partners (research) or participants (outreach)?*—in order to build trust and confidence, effective collaborations (within practitioner organisations and between practitioners and participants) are imperative (Table 1). Being a programme delivered by a network of organisations and working with a range of audiences, this was essential to the functioning of OPAL. Indeed it is important for all citizen science projects as they require the input not only of both scientists and participants but often a wide array of other partners too.

Firstly, is there enough *buy*-*in* from partners? Receiving adequate buy-in from all organisations involved can require considerable effort, time and resources (Table 1) yet failing to get the support from either the experts informing the project, the data end users, the outreach staff or the participants can create difficult working relationships and inadequate outputs. This was highlighted by one external collaborator who sat on an advisory committee for the OPAL Tree Health Survey and felt that buy-in from professional scientists was particularly key in “scientific disciplines where citizen science is new, novel or perceived as threatening”. It was also clear from the data that “the most effective projects are those that have a clear champion within the organisation taking part”. Recognising that partners are often stretched for time, OPAL assigned funding towards resources to support an effective balance between research and outreach by for example, funding PhD students to join the research labs of academics with the aim of freeing up time for these professional scientists to contribute to citizen science. When there are no leading supporters, relationships can break down, for example one collaborator noted “in our case, no one person was designated as leading on the OPAL-related work we were carrying out, and this may have reduced the impact of our work to other OPAL partners.” Champions are also very valuable in other parts of the project, from a strategic level (such as a member of a government department sitting on an advisory board) to a community level (such as an active member of the public coordinating group monitoring in a local park).

Secondly, how is *feedback* maintained between all stakeholders (partners, external organisations and participants)*?* The Community Scientist network was a key strength in the OPAL programme. These staff members provided an effective conduit between the in-house scientists and the community groups (Table 1). With diverse backgrounds in research, education and science communication, they were ideally placed for taking science into communities (as opposed to individuals coming to science centres like museums or universities). In addition to these staff, identifying key individuals who support the project’s work was found to be an effective way to spread messages throughout a community. Teachers for example, are gatekeepers to young people’s experience with science and as such OPAL created curriculum support for teachers by using the surveys to deliver their lessons. Participants could also undertake the surveys independently, and for this to be an effective experience for both learning and data collection in the absence of Community Scientist support, available resources needed to be clear, innovative and intellectually matched to the audience, and involve feedback to participants. In all surveys once participants had entered data, their results appeared on an interactive map so that they could compare their record to others and in some surveys participants could work out an instant score of environmental health (Table 1). This feedback benefit was clear as in the climate survey there was no such instant measure of quality so “although people liked the idea of contributing to a national data set many participants did not go away feeling they had learnt more about their local environment.” Intermediary results were also uploaded to the website to provide clear infographics displaying findings once data had been processed, and then lay summaries of scientific papers were also posted on the website. Forging collaborations between practitioners and participants can therefore be gained through effective communication.

#### C. Build a sufficiently diverse partnership (to widen scope)

It is important to tackle the third key trade-off—*jack of all trades, master of none?*—if scope and influence is to be initiated and expanded upon. There are many dimensions to a citizen science project and many sectors on which they can have an impact. In order to reduce the likelihood of scope and influence being compromised, we suggest that it is important to *build a sufficiently diverse partnership* (Table 1). The key to this is having “appropriate staff members to deliver complex projects” and diverse *expertise* where projects have broad aims. The OPAL programme was planned to bring together staff across research and outreach sectors. On the research side, academics based at English universities formed the regional project leaders, PhD students were employed to support regional research, and external personnel from government departments and environmental organisations sat on strategy boards and working groups. For outreach, museums, environmental educators and other public facing organisations formed part of the core partnership; and Communication Officers and Web Editors were employed for remote engagement. Sitting between the two sectors, the management team coordinate balancing the two aims and Community Scientists take science to the public with their expertise in science and experience in science communication. Participants also generate impact, i.e. through promoting “knowledge exchange rather than using citizens as mere suppliers of information” alongside enabling wider geographic and potentially more diverse areas to be studied.

Each of these stakeholders bring unique expertise to the partnership which contributes to its success at commandeering influence. Take Community Scientists for example, they: share their passion and knowledge (one noted “excitement about a tiny parasitic wasp or how fungi reproduce is what draws others in and brings it alive for them”); they integrate experience into resource development (one partner commented that they “bring their knowledge and experience of working with people of all backgrounds, ages and abilities to each individual survey”) and they understand about effective partnership working (one Community Scientist noted “working with amateur natural history societies requires a sensitive and responsive approach.”) While distinct roles were carefully planned, in reality most stakeholders pitched into many activities which yielded “unforeseen benefits of public engagement: First, [academic] staff developed and widened their communication and teaching skills by having to engage with new audiences (some of whom may not initially be interested in the subject). Second, we benefited from discussions with members of the public and hearing a wide range of views. Third, feedback strongly suggests that the public enjoyed and benefited from meeting scientists and being able to ask them questions.”

#### D. Target audience (to build social capacity)

In order to address the trade-off—*who is contributing and to what extent?*—within the field of social capacity it is helpful to understand who your *target audience* is and what their requirements are (Table 1). There is a spectrum of levels of involvement and sectors of society that citizen science projects work across and below we reflect on the latter.

Which *sectors of society* are considered*?* The OPAL portfolio aims to support ‘citizen science for everyone’ and has sought to provide multiple mechanisms for people to get involved, from taking a quick photograph of a Species Quest invertebrate through to undertaking a detailed hour long survey. Efforts were made by all staff to engage with groups that were classed as ‘hard to reach’. More than 129,000 beneficiaries were from these communities which included for example, those in areas of deprivation (as identified by the Index of Multiple Deprivation) or from black and ethnic minority (BME) communities through organisations such as Sheffield Black and Ethnic Minority Environmental Network. The primary message from OPAL staff in working with hard to reach groups was “know your audience”. For example, when working with BME communities (Table 1), one practitioner suggested that it may be useful to “identify BME community leaders and champions (e.g. from churches, mosques, temples and other places of worship) as an effective way to overcoming barriers to engagement and suspicion.” Of broader significance, when engaging BME groups in environmental work, it is important to recognise that some BME groups may feel excluded from the natural environment and experience a cultural severance (Table 1). To OPAL, reaching these sectors of society was fundamental, not only for the social benefits but also because participants from socio-economically deprived areas are under-represented in monitoring schemes [42] and because evidence suggests that deprived areas experience higher levels of environmental degradation [53]. Whoever the target audience is, they need to be understood.

Secondly, the mechanism through which participation can occur is important and as such, is *technology* integrated appropriately? Technology is a huge driver behind the current wave of citizen science. Within OPAL, technology enabled data to be captured, verified, stored, shared, transferred, analysed interpreted and understood and had the capacity to motivate and innovate. For example, comments highlighted that reaching some audiences—in particular young adults—was made easier by the greater use of technology and digital media which offered an inexpensive route in for citizen science practitioners to reach spaces already frequented by this audience (Table 1). Tools in the OPAL programme (Fig. 1) ranged from: an online identification tool—iSpot—which taps into a community of enthusiasts who support each other in verifying species identifications from photographs (390,000 observations received until mid-2014 of which 94 % received a determination [54]); to a digital learning suite—the OPAL Learning Lab—which provides guidance and fun online activities which mimic the Community Scientists role; to recording software—Indicia and iRecord—which enables communities to create local databases of interest to them (such as seaweed recording forms by the British Phycological Society to the Sea Life Tracker app by the organisation Nature Locator [55]). The Species Quest mobile app allowed participants to send photographs of species along with location and date information (key components of a biological record), which allowed scientists to subsequently verify the species identification and add positive record data points with confidence. The benefits of apps for high quality, rapid response geo-referenced records are clear and recommendations for design of smartphone applications can be found elsewhere [56], however, not all audiences (such as parts of natural history societies and older generations) have access to a computer/smartphone, or if they do, some may not be ‘tech-savvy’. Therefore digital communications were not solely relied upon. Indeed Mueller et al. [57] note that it is often those that may benefit most from new technologies that lose out. To provide a means for those without access to a computer to send in their results, a freepost address was set up shortly after the launch of the first OPAL survey [33]; this increased the amount of data returned and ensured that all those who wanted to participate (i.e. OPAL’s target audience) could do so. It is therefore essential to assess how technology can be used to maximum effect and where more traditional methods could be maintained. These questions are key considerations in order to understand whether communities are effectively supported to respond to social or ecological challenges.

## Evaluation framework: Can a project have its citizen science cake and eat it?

Is the proverb correct—is it impossible to ‘have it both ways’ in order to achieve two apparently conflicting objectives? Being a portfolio of projects aiming to balance research and outreach objectives, OPAL provided a lens through which to identify trade-offs and investigate mechanisms to work around these challenges in order to address this question. While OPAL is only one programme in a sea of other citizen science approaches, and some findings will be specific to OPAL, many other projects also aim to balance outreach and research. We therefore attempted to capture key lessons learned in the OPAL programme in order to investigate the practical approaches for overcoming trade-offs.

To illustrate how practical approaches can lead to a successful balance between research and outreach objectives, we have illustrated the findings from OPAL by combining them with the evaluation framework of Haywood and Besley [12, 45] (Fig. 2). We have taken learning points from the daily operations of the OPAL programme (which we term ‘Operational Indicators’) and matched them (slice A–D) with the factors that Haywood and Belsey suggest lead to a successful programme outcome (which we term ‘Outcome Indicators’). The first slice (A) proposes that through a project’s operations the outreach and research should be *fit for purpose* (through designing products to ensure quality products and monitoring quality throughout) with the end result that the *needs are met* of both scientists and participants. The second slice (B) proposes that *working collaboratively* (by getting adequate buy-in and obtaining feedback) is important when delivering multi-aim projects in order to build *trust and confidence* between the partners. The third slice (C) proposes that *building a diverse partnership* (will ensure expertise are available) in order to widen and advance the *scope* of the project. The last slice (D) suggests that considering the *target audience* (which sectors of society should be targeted and how technology should support this) is imperative when maximising *social capacity.*

OPAL received a relatively substantial budget with which to carry out its national scale operations which provided the resources for the network to explore the full spectrum of citizen science from outreach to research and evaluate the challenges and potential solutions along the way. While every project will have differing objectives and different levels of financial support, a number of common challenges are likely to be encountered. The solution to these will of course vary in detail but we suggest four broad operational approaches which may help to alleviate trade-offs encountered with dual-aim projects. Given the evidence presented within this manuscript we therefore believe that it may indeed be possible to have your citizen science cake and eat it, given appropriate planning, monitoring and adaptation.

## Abbreviations

BLF: Big Lottery Fund
BME: black and ethnic minority communities
CS: citizen science
OPAL: Open Air Laboratories
PES: public engagement in science
PUST: public understanding of science and technology
PPSR: public participation in scientific research
